# Coral reef structural complexity loss exposes coastlines to waves

**DOI:** 10.1038/s41598-023-28945-x

**Published:** 2023-01-30

**Authors:** Jérémy Carlot, Michalis Vousdoukas, Alessio Rovere, Theofanis Karambas, Hunter S. Lenihan, Mohsen Kayal, Mehdi Adjeroud, Gonzalo Pérez-Rosales, Laetitia Hedouin, Valeriano Parravicini

**Affiliations:** 1 PSL Université Paris, UAR 3278 CRIOBE—EPHE-UPVD-CNRS, 52 Av. Paul Alduy, 66000 Perpignan, France; 2 Laboratoire d’Excellence “CORAIL”, Paris, France; 3 CESAB—FRB, Montpellier, France; 4 Department of Marine Sciences, University of the Aegean, University Hill, 81100 Mytilene, Greece; 5 Centre for Marine Environmental Sciences (MARUM), Bremen, Germany; 6 Dipartimento di Scienze AmbientaliInformatica e Statistica (DAIS), Ca’ Foscari University of Venice, Venice, Italy; 7 Department of Civil Engineering, Aristotle University of Thessaloniki, Thessaloniki, Greece; 8 Bren School of Environmental Science and Management, University of California, Santa Barbara, USA; 9 9ENTROPIE, IRD, Université de la Réunion, CNRS, IFREMER, Université de la Nouvelle-Calédonie, Nouméa, New Caledonia; 10 ENTROPIE, IRD, Université de la Réunion, CNRS, Perpignan, France; 11 PSL Université - EPHE-UPVD-CNRS, UAR 3278 CRIOBE, Papetoai, French Polynesia

**Keywords:** Ecological modelling, Ecosystem services, Physical oceanography, Environmental health

## Abstract

Coral reefs offer natural coastal protection by attenuating incoming waves. Here we combine unique coral disturbance-recovery observations with hydrodynamic models to quantify how structural complexity dissipates incoming wave energy. We find that if the structural complexity of healthy coral reefs conditions is halved, extreme wave run-up heights that occur once in a 100-years will become 50 times more frequent, threatening reef-backed coastal communities with increased waves, erosion, and flooding.

## Introduction

By March 2020, the world’s human population grew to more than 7.8 billion people^1^, with most of the growth taking place in coastal areas where population density is highly concentrated^2^. As a result, the number of people exposed to intense coastal storms and flooding also markedly increased^3^. Climate change is expected to further exacerbate the risks of flooding due to rising seas^4,5^ and changes in weather patterns^6–8^. In response, societies are called to design effective coastal protection schemes^9^, which preserve or strengthen the current natural protection offered in the form of ecosystem services^10^.

This is particularly true in tropical areas, where more than 500 million people are protected by coral reefs^11^. Shallow water coral reefs act as natural breakwaters^12^, and can attenuate up to 98% of the incoming wave energy^13^. However, coral reefs are being rapidly degraded by intensifying anthropogenic stressors, including ocean acidification, rising seas, pollution, and sedimentation^14^. Such perturbations usually reduce living coral cover and subsequently habitat structural complexity, thereby leading to reef flattening, a process observed worldwide^15^. Such trends reduce the natural protective capacity of reefs, further intensifying coastal flood and erosion risks^9,16^. Although the link between wave attenuation and reef structural complexity is widely accepted among scientists in theory, quantitative examinations of the protective capacity of reefs under real world conditions as anticipated for the near future are yet limited^17,18^. Indeed, most of the studies investigated the wave energy dissipation in conditions where coral reefs would be entirely wiped out or not^12,19^, while others chose a wider geomorphology-based approach exploring flooding risk as a function of reef proximity or geologic structure^20–22^.

Here we report field observations and numerical models to quantify the natural protection offered by coral reefs at a high-energy site in Mo’orea, French Polynesia. First, we monitor changes in coral community abundance and size-distribution over a 10-year full cycle of disturbance and recovery (2005–2016), and quantify the associated reef structural complexity (see "Materials and methods" for details). Second, we use in situ hydrodynamic measurements to link the structural complexity levels with the wave energy dissipation capacity of the reef, and to calibrate a Boussinesq wave propagation model, which simulates the interplay between hydrodynamics and reef topography on the site. Third, we apply a probabilistic framework that assesses a coral reef’s capacity to attenuate incoming wave energy, applying our model under all possible wave and structural complexity conditions for the study site. We evaluate the resulting 10,000 runs by using as proxies wave overwash, impact, and flooding, the significant wave run-up, and finally, the 2% exceedance wave run-up height (R_2%_).

## Materials and methods

### Ecological sampling and structural complexity profiles

**Figure 1:**
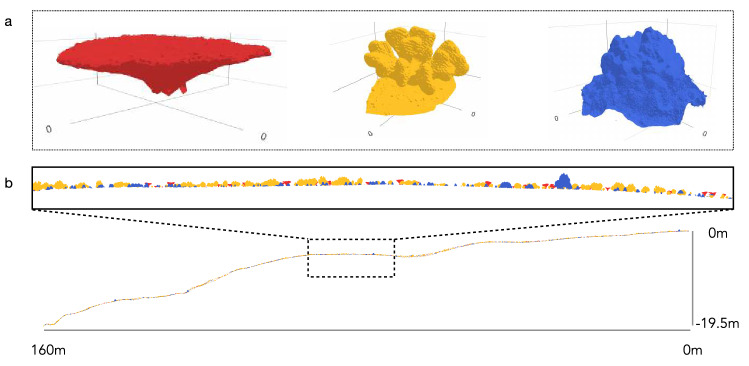
(**a**) Representation of the three different coral species (*Acropora hyacinthus* in red, *Pocillopora *cf. *verrucosa* in yellow, and *Porites lutea* in blue). (**b**) A representaitive Ha’apiti reef cross-section simulation (one of 1000 total simulations) on the outer slope across a water depth range of 0–20 m.

The ecological sampling consists of 10 surveys, taking place in 2005 and from 2008 to 2016, and documents changes in coral colony abundance and size distributions (i.e. width, length, and height) for the three most conspicuous taxa (i.e.* Acropora*, *Pocillopora*, and *Porites*) within a 10 m^2^ transect on the outer slope^23^. To quantify reef structural complexity, we built a 3D model of the coral assemblages distributed along a cross-section of the reef substrate separating the 20 m water depth from the reef crest, representing a 160 m stretch along the reef slope (Fig. 1). First, we take 200 overlapping high-resolution photos (300 dpi) of 10 individual corals from each species (i.e. n = 30 coral colonies) and built 3D models using the Agisoft Metashape software^24^, capturing intra- and inter-species morphological variability (Fig. 1). Then, we systematically and randomly select one of the ten 3D coral models for each taxon to add to the substrate until that the sum of the planar area for each 3D coral models match with the coral cover reported for each taxon and for each year^23^. We randomly place coral colonies along the 160 m reef cross-section going from 20 m depth to the reef crest (Fig. 1). The individual coral 3D models are resized in width, length, and height according to ecological surveys, and, randomly rotated between − π/2 and π/2 to ensure ecological variability. Finally, we estimated structural complexity of the 3D coral assemblage model using the function *rumple_index* of the *LidR package*^25^ in R 4.0.0^26^. We repeat this approach 100 times for each year, resulting in a total of 1000 reef structural complexity profiles. Our estimates are consistent with previous reef structural complexity estimates at this location^27^.

### Hydrodynamic and topographic measurements

**Figure 2:**
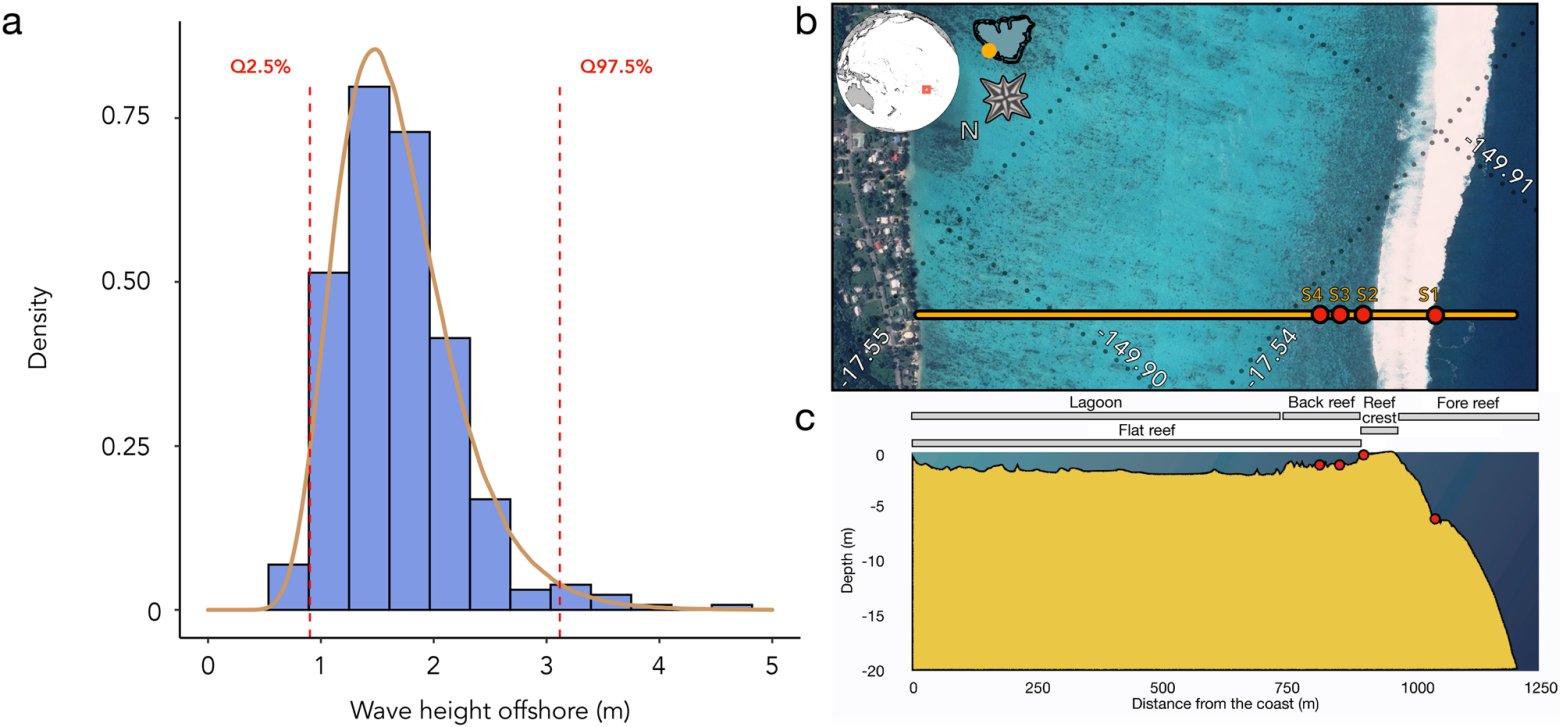
(**a**) Histogram of the offshore wave height (m) at Ha’apiti, Mo’orea (French Polynesia) in 2016. (**b**) Aerial view of Ha’apiti (WorldView-3 imagery) with an outline of the wave transect and sensor location. The ecological sampling took place near the S1 location c. Topographic cross-section of the wave transect and position of the sensors on the sea bottom.

Mo’orea (French Polynesia) is encircled by coral reefs, 500–700 m wide with a dominant swell direction coming from the southwest. In this study, we focus on Ha’apiti, a site with a southwest orientation that is considered as a high-energy site^28^. We extract 30-year offshore wave data (1980–2010) from a wave hindcast^8,29^ (Fig. 2a). We also collect high-frequency, in situ wave data using INW PT2X Aquistar and DHI SensorONE pressure transducers (PTs), which are logged at 4 Hz^30^. The sensors are installed at four locations along a cross-shelf gradient (Fig. 2b,c) covering a 250 m long stretch, including sections through the fore reef, reef crest, and reef flat. Pressure records are corrected for pressure attenuation with depth^31^ and are split into 15-min bursts^30^.

The beach profile and the reef morphology are measured using airborne bathymetric and topo-bathymetric lidar surveys conducted in June 2015 by the Service Hydrographique & Océanographique de la Marine (SHOM). The bathymetric data are defined by the combination of bathymetric laser (for the submerged part of the beach) and topo-bathymetric laser (for the subaerial beach). The data come at 1 m resolution and are available at https://diffusion.shom.fr.

### Hydraulic roughness vs structural complexity

**Figure 3:**
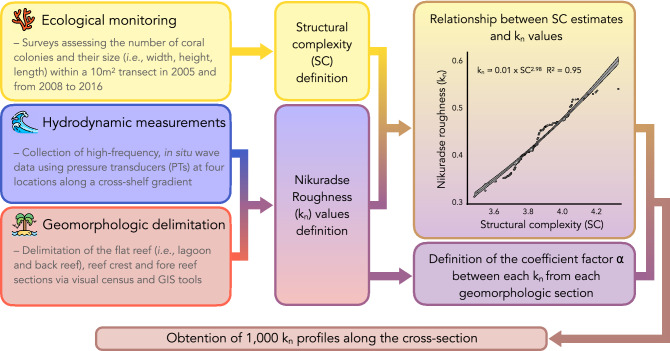
Flow chart illustrating how the k_n_ profiles have been obtained along the cross-section at Ha’apiti. The relationship between the Structural complexity (SC) and the Nikuradse roughness (k_n_) measurements can be described as k_n_ = 0.01 × SC^2.98^.

Spectral attenuation analysis of the water level measurements^32,33^ is used to estimate the Nikuradse (hydraulic; k_n_) roughness^34^ of the coral reef surface along the beach profile sections covered by the pressure transducers. The method is described in detail in the references provided above and uses the conservation of energy equations to obtain estimates of wave energy dissipation from friction. We obtain more than 300 k_n_ estimates for each pair of sensors, each representing a different geomorphologic section. Since the field measurements took place in 2015, the k_n_ outputs obtained from the fore reef section concur with the reef structural complexity estimates of that year (Fig. 3). Then, we define a coefficient factor according to the geomorphologic section as ⍺_back reef_ = k_n, back reef_/k_n, fore reef_ and ⍺_reef crest_ = k_n, reef crest_/k_n, fore reef_. We carefully delineate the sandy section from the reef sections within the cross-shelf gradient (i.e. within the reef flat, lagoon section) and apply the following procedure. First, for the reef sections, we apply the relationship between the reef structural complexity and k_n_ (Fig. 3) to convert our reef structural complexity estimates into continuous k_n_ profiles through Monte Carlo simulations, using the coefficient factor of each geomorphologic section (*e.g.,* forereef, reef crest, and back reef). Second, for the sandy section, we define k_n_ on the grounds of the mean grain size (d_50_ = 63 μm). Applying this workflow (Fig. 3), we obtain 100 continuous k_n_ profiles for each year (i.e. n = 1000 k_n_ profiles in total).

### Hydrodynamic model

Nearshore wave propagation is simulated using a nonlinear wave model based on the Boussinesq Equations^35^. The rationale of using a Boussinesq type model instead of other types of models (e.g. SWAN) is that the former is able to describe in detail (i.e. 1 m grid resolution) several hydrodynamic parameters (e.g. nearshore nonlinear wave propagation, shoaling, refraction, dissipation due to the bottom friction and breaking and run-up) in the swash zone. The model is defined as follows:

1 $$\frac{\partial U}{\partial t}+\frac{1}{h}\frac{\partial {M}_{u}}{\partial x}-\frac{1}{h}U\frac{\partial \left(Uh\right)}{\partial x}+g\frac{\partial\upzeta }{\partial x}=\frac{\left({d}^{2}+2\partial\upzeta \right)}{3}\frac{{\partial }^{3}U}{\partial {x}^{2}\partial t}+{d}_{x}h\frac{{\partial }^{2}U}{\partial x\partial t}+\frac{{\partial }^{2}}{3}\left(U\frac{{\partial }^{3}U}{{\partial x}^{3}}-\frac{\partial U}{\partial x}\frac{{\partial }^{2}U}{\partial {x}^{2}}\right)+d\frac{\partial\upzeta }{\partial \mathrm{x}}\frac{{\partial }^{2}U}{\partial\upzeta \partial \mathrm{t}}+d{d}_{x}U\frac{{\partial }^{2}U}{\partial {x}^{2}}+{d}_{x}\frac{\partial\upzeta }{\partial \mathrm{x}}\frac{\partial \mathrm{U}}{\partial \mathrm{t}}-d\frac{{\partial }^{2}}{\partial \mathrm{x}\partial \mathrm{t}}\left(\delta \frac{\partial \mathrm{U}}{\partial \mathrm{x}}\right)+E-\frac{{\tau }_{b}}{\rho h}+B{d}^{2}\left(\frac{{\partial }^{3}U}{\partial {x}^{2}}+g\frac{{\partial }^{3}\upzeta }{\partial {x}^{3}}+\frac{{\partial }^{2}\left(U\frac{\partial U}{\partial x}\right)}{\partial {x}^{2}}\right)+2Bd{d}_{x}\left(\frac{{\partial }^{2}U}{\partial x\partial t}+g\frac{{\partial }^{2}\upzeta }{\partial {\mathrm{x}}^{2}}\right),$$

where, *U* is the mean over the depth horizontal velocity, ζ is the surface elevation, d is the water depth, u_o_ is the near bottom velocity, *h* = *d* + ζ, ${M}_{u}=\left(d+\zeta \right){u}_{0}^{2}+\delta ({c}^{2}-{u}_{0}^{2})$, δ is the roller thickness determined geometrically^36^, E is an eddy viscosity, *τ*_*b*_ is the bed friction term and *B* = 1/15^35^.

In this work the wave breaking mechanism is based on the surface roller concept^36^. However, in the swash zone, surface roller is not present and the eddy viscosity concept is used to describe the breaking process. The term E in Eq. (1) is written:

2 $${\mathrm{E}}_{{\mathrm{b}}_{\mathrm{x}}}= {\mathrm{B}}_{\mathrm{b}}\frac{1}{\mathrm{h}+\upeta }{\left\{{{\mathrm{v}}_{e}\left[\left(\mathrm{h}+\upeta \right)\mathrm{U}\right]}_{\mathrm{x}}\right\}}_{\mathrm{x}},$$

where ${v}_{e}$ is the eddy viscosity coefficient:

3 $${\mathrm{v}}_{\mathrm{e}}={{\ell}}^{2}\left|\frac{\partial {\mathrm{U}}}{\partial {\mathrm{x}}}\right|,$$

where ${\ell}$ is the mixing length ${\ell}$ = 3.5 h και Β_b_^37^.

The width of the swash zone is assumed to extend from the run-down point (seaward boundary) up to the run-up point (landward boundary). We start from a first estimate of the run-up R using the Stockdon formula^38^ and the depths below R/4 are considered as the swash zone, using Eq. (2). The final wave run-up height R which comes as output is estimated by the model.

The ‘dry bed’ boundary condition is used to simulate run-up^35^. The numerical solution is based on the fourth-order time predictor–corrector scheme^39^. Therefore, the bed friction term *τ*_*b*_ is calculated such as:

4 $${\tau }_{bx}=\frac{1}{2}\rho {f}_{w}U\left|U\right|,$$

where *f*_*w*_ is the bottom friction coefficient^40^, which is an explicit approximation to the implicit, semi-empirical formula given by Jonsson, 1967^41^.

5 $${f}_{\mathrm{w}}=\mathrm{exp}\left[{5.213\left(\frac{{\mathrm{k}}_{\mathrm{n}}}{{\mathrm{\alpha }}_{0}}\right)}^{0.194}-5.977\right],$$

**w**here α_o_ is the amplitude of the near-bed wave orbital motion and k_n_ is the Nikuradse roughness height.

### Simulations and post processing

We use our wave propagation model to assess how different coral reef states affect the impact waves have on the coast. We run an ensemble of 10,000 simulations that covers all the possible combinations of (i) 10 bottom roughness profiles expressing the different observed coral reef states (i.e. healthy *vs.* not unhealthy); and (ii) 1000 percentiles of wave conditions. The wave conditions are produced as follows: (i) from the weekly values, we estimate all significant wave height (H_s_) percentiles from 0.1 to 100, with a step of 0.1; (ii) the resulting 1000 H_s_ values are linked to the corresponding peak wave period T_p_ using a copula expressing the dependence of the two variables^42^. The output of the simulations is the nearshore H_s_ and 2% exceedance run-up (R_2%_) height for each of the 1000 conditions and 10 coral reef states. To quantify how the coral reef states are altering wave propagation during extreme events, we apply extreme value analysis to estimate the R_2%_ for different return periods^43^. We then compare how the return period curves changed from the two coral reef states and we define the change in frequency of extreme R_2%_ under unhealthy coral reefs. It is important to highlight that the tidal range is < 20 cm at our study site, so will have negligible effect compared to other parameters. In systems with higher tidal range, tidal water level fluctuations should be also considered in the probabilistic framework.

Using the outputs of our simulations, we develop a Bayesian model which includes all the interdependencies between the run-up, the significant offshore wave height (H_s, offshore_), and the reef structural complexity (SC). Our model is built in the R package *brms*^44,45^ and follows the following structure:

$$RU \sim \mathcal{N}\left({\mu }_{s}, \sigma \right),$$

$${\mu }_{s}=\left(\alpha + {\sigma }_{\zeta }\right)\times {H}_{s, offshore}+\left(\beta + {\sigma }_{\zeta }\right) \times SC+\left(\gamma + {\sigma }_{\zeta }\right) \times SC:{H}_{s, offshore},$$

$$\alpha \sim \mathcal{N}\left(0, 1\right); \beta \sim \mathcal{N}\left(0, 1\right); \gamma \sim \mathcal{N}\left(0, 1\right); \sigma \sim\Gamma \left(\mathrm{2,0.1}\right); {\sigma }_{\zeta } \sim\Gamma \left(\mathrm{2,0.1}\right),$$

where RU is the run-up (m), SC is the structural complexity, and H_s,offshore_ is the significant offshore wave height (m). The prior sampling is specified to follow a Gaussian ($\mathcal{N}$ (location, scale)) and a Gamma (Γ(shape, inverse scale)) distribution. We ran our models with four chains, 5000 draws per chain, and a warm-up period of 1000 steps, thus retaining 16,000 draws to construct posterior distributions. We verify chain convergence (n = 4) with trace plots and confirm that R_hat_ (the potential scale-reduction factor) is lower than 1.05^46^. Drawing on our model, we predict the run-up according to six offshore wave height conditions (i.e. from 1 to 6 m with a step of 1 m) and the structural complexity gradient at Mo’orea from 2005 to 2016. In order to define the residual run-up height according to H_s, offshore_ and the reef structural complexity, we subtract from our run-up estimates the minimum value obtained for each offshore wave height condition.

## Results and discussion

**Figure 4:**
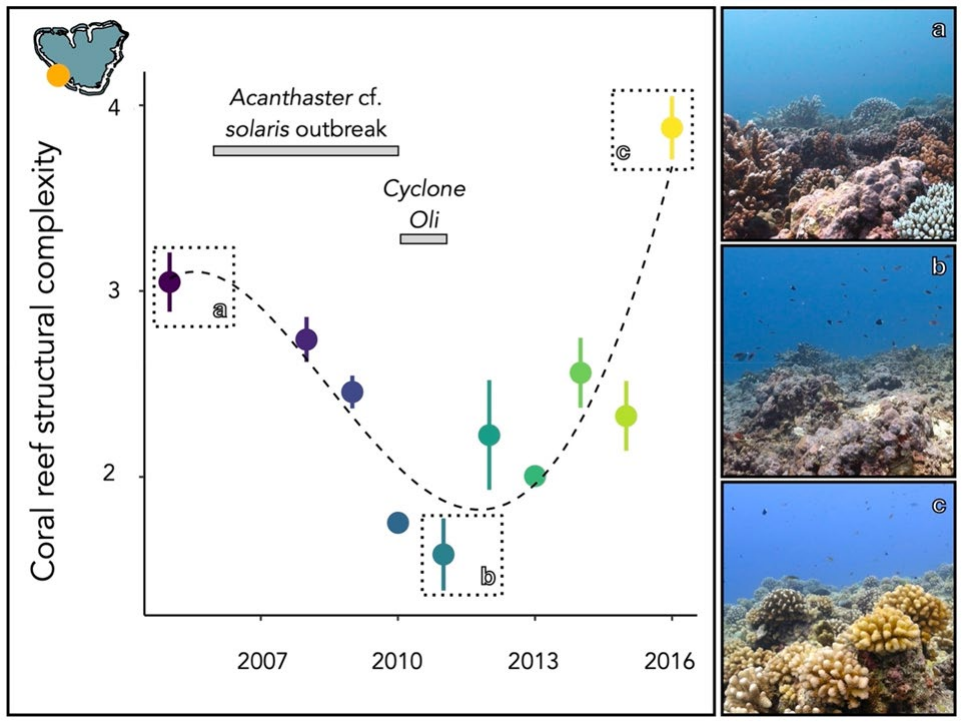
Evolution of the structural complexity from 2005 to 2016 on the west side of Mo’orea (French Polynesia). Perturbations included a predatory sea star (*Acanthaster* cf. *solaris*) outbreak from 2006 to 2009 and a cyclone in February 2010. Photographs illustrate the reefscapes in (**a**) 2005, (**b**) 2011 (unhealthy reef) and (**c**) 2016 (healthy reef).

Between 2006 and 2010, Mo’orea experienced an outbreak of the predatory sea star *Acanthaster* cf. *solaris* and a cyclone reducing consequently the living coral cover from 50 to 3% and halving the overall structural complexity (Fig. 4). Accordingly, we consider the year after these disturbances (i.e., 2011) as unhealthy coral reefs. By the year 2016, coral cover recovered from these disturbances with a dominance of *Pocillopora* cf. *verrucosa*, presenting a higher complexity profile from the three coral species and increasing the overall reef structural complexity (Fig. 4). Therefore, we consider the year 2016 as a healthy coral reef in terms of structural complexity. Overall, the median structural complexity from unhealthy reefs is 1.58 with the very likely range of 1.25–1.92 (5th–95th percentile), whereas the median structural complexity from healthy reefs is 3.86 with the very likely range of 3.59–4.20.

**Figure 5:**
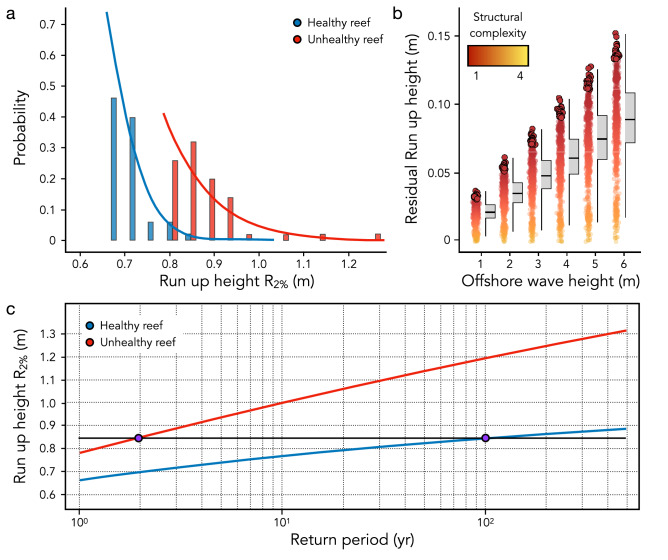
(**a**) Comparisons of the 2% exceedance wave run-up height under healthy (blue) and unhealthy (red) coral reefs conditions. For each case, we superimpose the fitted Generalized Pareto Distribution (lines) on the empirical probability density function (histograms). (**b**) Residual run-up height according to the structural complexity loss and the magnitude of the event. The black-circled points highlight the 2% exceedance wave run-up height. (**c**) Extreme 2% exceedance wave run-up height (R_2%_) under healthy (blue) and unhealthy (red) coral reefs conditions. The curves show how R_2%_ varies with the return period, while the black horizontal line highlights that the one in a 100-year R_2%_ under healthy coral reef conditions (onshore wave run-up of 0.85 m) will occur at least every two years if coral reefs deteriorate.

Field measurements show that the median Nikuradse (hydraulic; k_n_) roughness^34^ of healthy reefs at Mo’orea is 0.42, with a very likely range of 0.34–0.52. We combine the simultaneous k_n_ and structural complexity measurements in Monte Carlo simulations, in order to produce continuous k_n_ profiles for all observed coral reef states, which we use in our wave propagation simulations. In line with previous studies^13^, our modeling results show that coral reefs absorb 77% to 91% of the incoming wave energy, with the range expressing the variability of wave and structural complexity conditions. We also confirm the previously reported strong correlation between structural complexity and wave dissipation^28^, and hence the reduction of wave run-up height on the coast (Fig. 5a). Unhealthy reef conditions result in wave run-up heights 9.6% higher compared to healthy ones (very likely range 8.7–10.7%). Our results also show that wave run up height reduction increases with wave height, highlighting that coral reef protection is more important when it matters most; i.e. during extreme events (Fig. 5b). For example, structural complexity has twice the potential to dissipate offshore waves with significant wave height H_*s*_ = 3 m (exceeding the 97.5th percentile in our study site) compared to a wave with H_*s*_ = 1 m (below the 2.5th percentile, Fig. 2a).

We apply an extreme value analysis to assess how the loss of coral reef structural complexity controls the frequency of extreme wave run-up. Our findings show that the frequency of extreme overwash and flood conditions on the shore will increase as long as coral health deteriorates, even without rising sea-levels. For example, wave run-up heights, which under healthy reef conditions occur once in a 100-year, will become 50 times more frequent if coral reef structural complexity deteriorates (Fig. 5c). This effect is further amplified with the increase in the return period of big-wave events; a 10-year event will become *ca.* 10 times more frequent, whereas a 500-year event will become at least 150 times more frequent.

Coral reefs thrive mainly in tropical and sub-tropical regions where the highest intensification of extreme sea levels (ESLs) has been projected^8^; i.e. the 100 year ESL will occur at least every 10 years after 2050, every year in many locations. Our results imply that reef degradation will leave reef-supporting coastlines more exposed to coastal flooding and erosion than previously projected, thereby exacerbating the risks associated with sea level rise. Wave attenuation relies mainly on reef accretion and structural complexity^28^, for which the projections are not encouraging. Under a high greenhouse gas emissions scenario by the year 2050, 94% of the reefs worldwide may cease to accrete and start to flatten due to ocean acidification and warming^47^ as it is already the case in other regions, including Florida^48^ and some Caribbean islands^49^. Thus, the effects of rising seas are expected to be further amplified by the loss of living corals, painting a grim picture for the future safety of tropical coastal societies and highlights the critical importance of emission mitigation and coral preservation efforts.

## Data Availability

Code and data are available at https://github.com/JayCrlt/Wave_energy.git.
